# Sex-Based Contributors to and Consequences of Post-traumatic Stress Disorder

**DOI:** 10.1007/s11920-023-01421-z

**Published:** 2023-05-10

**Authors:** Lucy V. Hiscox, Tamsin H. Sharp, Miranda Olff, Soraya Seedat, Sarah L. Halligan

**Affiliations:** 1 Department of Psychology, University of Bath, Bath, UK; 2 Department of Psychiatry, Amsterdam University Medical Centers, Academic Medical Center, University of Amsterdam, Amsterdam, the Netherlands; 3 ARQ National Psychotrauma Centre, Diemen, the Netherlands; 4 Department of Psychiatry, Faculty of Medicine and Health Sciences, Stellenbosch University, Cape Town, South Africa; 5 SAMRC Unit on Risk & Resilience in Mental Disorders, Department of Psychiatry and Mental Health, University of Cape Town, Cape Town, South Africa

**Keywords:** PTSD, Sex differences, Trauma, Sex characteristics, Post-traumatic stress

## Abstract

**Purpose of Review:**

Women are twice as likely to develop post-traumatic stress disorder (PTSD) compared to men after a traumatic experience. The purpose of this mini review was to explore recent research on biological contributors to this sex difference.

**Recent Findings:**

We identified 51 studies published since 2019. Studies found that beyond the influence of sex on the prevalence and symptoms of PTSD, there is evidence for and against sex-based differences in genetic and epigenetic factors (*n* = 8), brain structure and function (*n* = 11), neuroendocrine and inflammatory responses (*n* = 5), and in the role of sleep on emotional memory processing (*n* = 1). Sex differences were also observed in recovery and during PTSD treatment (*n* = 16). Finally, there is emerging evidence of sex-differentiated risk for medical and psychiatric comorbidities in PTSD (*n* = 10).

**Summary:**

Rapid advances are being made using integrated multidisciplinary approaches to understand why females are at a heightened risk for developing PTSD.

## Introduction

Biological sex influences risk of post-traumatic stress disorder (PTSD) following trauma exposure through potentially complex and varied biological and non-biological (e.g. psychosocial and environmental) mechanisms. PTSD is two to three times more common in women than in men [1, 2]. This sex bias likely emerges in adolescence [3, 4] and is attenuated in older age [5]. As well as differing prevalence rates, men and women also differ in the types of symptoms reported, with women reporting higher levels of intrusive and avoidance symptoms compared to men [6]. Historically, hormonal factors have been emphasised as one of the most potent putative mechanisms for the sex differentiation in PTSD. However, the last decade has seen a rapid increase in our understanding of possible sex differences in genetic susceptibility and resilience to development of PTSD after trauma [7••]. Over the past 3 years, there has also been increasing research attention on a range of other biological contributory factors and new evidence has emerged in relation to sex differences in treatment response to and recovery from PTSD. We synthesise the state of this evidence in this mini review focusing on the evidence for sex differences in (1) genetic and epigenetic factors; (2) other biological factors including (i) brain structure and function, (ii) immunological, neuroendocrine, and neuropeptide responses, and (iii) the role of sleep; (3) rates of recovery and response to treatment; and (4) medical and psychiatric comorbidities, in PTSD.

## Methods

Database searches were conducted in PubMed and PsychINFO for peer-reviewed journal articles examining potential contributors to variance in PTSD, focusing specifically on biological sex-based influences. Search terms included “PTSD” OR “posttraumatic stress disorder” AND “sex” OR “sex characteristics” OR “sex differences”. Studies focusing on gender roles or non-biological contributors to PTSD were not included, as this area has been recently covered elsewhere [8]. Studies also needed to have included both a biological male and female group; non-binary subjects were not included. Filters included “Humans” and searches were carried out from 2019 to December 2022. A total of 1533 articles were identified which were then screened for eligibility though title or abstract screening. The majority of articles were excluded at this stage (*n* = 1418). Only articles including human subjects (> 18 years) exposed to trauma and that examined the effects of sex related influences on PTSD were included. Of the remaining 115 articles, a further 64 were excluded after reading the full text, including review article(s), preclinical animal models of PTSD, non-biological contributors to PTSD (i.e. the role of gender); prevalence and symptoms of PTSD, or studies of children/adolescents. An overview of the 51 included studies is presented in Table 1 with specific studies highlighted in the sections that follow.

**Table 1:** Characteristics of studies included in the review

**Study**	**Measurement**	**Population**	**N**	**Study design**	**Trauma type**	**Country**
**Genetic and epigenetic factors (*n* = 8)**						
Nievergelt et al. (2019) [9]	Genome-wide association	PTSD and a mix of trauma exposed and non-trauma exposed controls	PTSD = 32,428 Controls = 174, 227	CS (GWAS)	Mixed	60 sites worldwide including European, Latino/Hispanic and South African populations
Wendt et al. (2021) [10]	Multivariate gene-by-environment genome-wide interaction study	Population-based cohort	123,633	CS (GWEIS)	Mixed	UK
Girgenti et al. (2020) [11]	Differential gene expression and network analyses	PTSD and controls	PTSD = 52 Controls = 46	CS	Mixed	USA
Stone et al. (2020) [12]	Gene co-expression network analysis	PTSD and controls	PTSD = 52 Controls = 46	CS	Mixed	USA
Nawijn et al. (2019) [13]	Oxytocin receptor gene methylation levels	Police officers with and without PTSD	PTSD = 31 Trauma exposed controls = 36	CS	Mixed	The Netherlands
Kim et al. (2019) [14•]	DNAm- based leukocyte composition	Participants from the Detroit Neighbourhood Health Study and Grady Trauma Project	483	CS	Mixed	USA
Huckins et al. (2020) [15]	Transcriptome-wide association	PTSD and a mix of trauma exposed and non-trauma exposed controls	PTSD = 29,539 Controls = 166,145	CS (TWAS)	Mixed	60 sites worldwide including European, Latino/Hispanic and South African populations
Muniz Carvalho et al. (2021) [16]	Genetic correlation, bidirectional causal inference analysis	Summary statistics derived from PGC-PTSD, GIANT consortium, UK Biobank and the Million Veteran Programme	N/A	Genetic correlation and Mendelian Randomization	Mixed	Summary statistics derived from European and African populations
**Brain structure and function (*n* = 11)**						
Roeckner et al. (2022) [19•]	Subcortical volumes and cortical thickness	Trauma survivors who may go on to develop PTSD	93	L	Mixed	USA
Sun, Rakesh, Haswell, et al. (2022) [20]	Cortical thickness	PTSD and trauma-exposed controls	PTSD = 1340 Controls = 2057	CS	Mixed	29 sites located on 5 continents
Sun, Rakesh, Clarke-Rubright et al. (2022) [21]	Cortical thickness (CT) and surface area (SA) networks	PTSD and trauma-exposed controls	PTSD = 1344; Controls: 2073	CS	Mixed	29 sites located on 5 continents
Clausen et al. (2022) [22]	Brain age	PTSD and trauma-exposed controls	PTSD = 882 Controls = 1347	CS	Mixed	21 sites located on 5 continents
Dennis et al. (2021) [24]	White matter microstructure	PTSD and trauma-exposed controls	PTSD = 1426 Controls = 1621	CS	Mixed	28 sites located on 5 continents
Suo et al. (2021) [25]	White matter microstructure	PTSD and trauma-exposed controls	PTSD = 77 Controls = 76	CS	Earthquake survivors	China
Averill et al. (2022) [26]	Glutamatergic synaptic strength in prefrontal cortex	PTSD and controls	PTSD = 16 Controls = 18	CS	NA	USA
Luo, Qi et al. (2019) [27]	Whole-brain network topology	PTSD, lost-only-child non-PTSD parents (NPTSD), and normal controls	PTSD = 51 NPTSD = 93 Controls = 50	CS	Loss of their only child	China
Luo, Liu et al. (2021) [28]	Functional connectivity of hippocampal subfields	PTSD, lost-only-child non-PTSD parents (NPTSD), and normal controls	PTSD = 55 NPTSD = 30 Controls = 50	CS	Loss of their only child	China
McGlade et al. (2020) [29]	Functional connectivity of amygdala subnuclei	PTSD or MDD	38	CS	Combat/military	USA
Sippel et al. (2021) [30]	Intranasal oxytocin on functional connectivity	PTSD and controls	PTSD = 16 Controls = 18	CS	Childhood trauma	USA
**Immunological and neuroendocrine responses (*n* = 5)**						
Lalonde et al. (2021) [32•]	Inflammatory cytokines and steroid hormones	Trauma survivors who may go on to develop PTSD	376	L	Criterion A trauma	USA
Küffer et al. (2019) [33]	Inflammatory cytokines	PTSD and controls	PTSD = 44 Controls = 49	L (overnight)	Mixed	USA
Bartholomew et al. (2022) [34]	Hormonal contraceptives (HC) on fear conditioning and extinction	Trauma exposed women on and off HC and aged-matched men with a range of PTSD symptom severity	Men = 48 Women on HC = 13 Women off HC = 20	L	Mixed	USA
Carmassi et al. (2021) [35]	Plasma oxytocin levels	PTSD and controls	PTSD = 26 Controls = 26	CS	Mixed	Italy
Tural and Iosifescu (2020) [36]	Neuropeptide Y levels	9 eligible studies	PTSD = 144 Controls = 308	CS	Mixed	Meta-analysis
**Role of sleep (*n* = 1)**						
Richards et al. (2022) [39]	REM sleep effects	PTSD +, PTSD-, and PTSD remission	PTSD + = 15 PTSD- = 13 PTSD remission = 18	L	Criterion A trauma	USA
**Treatment/recovery (*n* = 16)**						
Diamond et al. (2022) [40•]	Change in PTSD prevalence over 2 years	78 eligible studies	16,484	L	Mixed	Meta-analysis
van Zuiden et al. (2022) [41]	Symptom trajectories over 1 year	Emergency department patients	554	L	Mixed	The Netherlands
Stefanovics et al. (2022) [42]	Natural diagnostic remission	Lifetime PTSD patients in general population	1997	L	Mixed	USA
Lee et al. (2020) [43]	20-year course of symptoms	Operations Enduring Freedom and Iraqi Freedom veterans	1353	L	Combat/military	USA
Holder et al. (2020) [44]	Effect of prolonged exposure (PE) therapy	Iraq and Afghanistan war veterans	10,234	L	Combat/military	USA
Khan et al. (2020) [45•]	Effect of cognitive processing therapy (CPT) or PE	All post 9/11 veterans who had a PTSD diagnosis from 10/2001–9/2017	9711	L	Combat/military	USA
Stefanovics and Rosenbeck (2020) [46]	Effect of specialised intensive VHA PTSD treatment	Veterans	3370	L	Combat/military	USA
Scharff et al. (2022) [47]	Effectiveness of standardized care package	PTSD outpatients	948	L	Mixed	Denmark
Glassman et al. (2020) [48]	Predictors of quality of life following cognitive processing therapy	Veterans and civilians with PTSD	251	L	Mixed	USA
Shiner et al. (2021) [49]	Response to medications (e.g. fluoxetine, paroxetine)	VA outpatients	6839	L	Combat/military	USA
Nøhr et al. (2021) [50]	Effect of paroxetine or sertraline	Participants from clinical trial	390	L	Mixed	Multi-country
Lipov et al. (2022) [51]	Effect of stellate ganglion blockage	Civilian and military patients with PTSD	327	L	Mixed	USA
Valenstein-Mah et al. (2019) [52]	Initiation and completion of psychotherapy	Veterans	7218	L	Combat/military	USA
Harper et al., (2022) [53]	PTSD symptom severity in mental health service use	Veterans	1200	L	Combat/military	USA
Hinton et al. (2022) [54]	Predictors of drop-out from PTSD treatment	Veterans and first responders	95	L	Combat/military	USA
Hadlandsmyth et al. (2022) [55•]	Medication prescribing patterns	Veterans receiving treatment for PTSD	877,785	CS	Combat/military	USA
**Medical comorbidities *(n* = 10)**						
Rosman et al. (2019) [59]	Risk of stroke	Veterans free of TIA and ischemic stroke at baseline	PTSD: 282,382 Controls: 705,473	L	Combat/military	USA
Seligowski et al. (2021) [60]	Posttraumatic autonomic functioning	Emergency department patients	192	L	Index trauma: motor vehicle collision	USA
LIhua et al. (2020) [61]	Risk of metabolic syndrome	PTSD and traumatised non-PTSD controls	PTSD = 201 Controls = 2666	CS	Mixed	China
Holmstrup et al. (2020) [62]	Subclinical cardiovascular disease	Young adults	61	CS	NA	USA
Gunak et al. (2020) [58••]	Risk of dementia	PTSD and controls	PTSD = 89,493 Controls = 1,604,185	L	Mixed	Meta-analysis
Barer et al. (2022) [64]	Risk of Parkinson’s disease	PTSD and controls	PTSD = 8336 Controls = 8336	L	Mixed	Israel
Jiang et al. (2019) [65]	Risk of incident infections	PTSD and controls	PTSD = 4984 Controls = 24,920	L	Mixed	Denmark
Walter et al. (2022) [66]	Psychiatric comorbidities	Service members with PTSD	9447	CS	Combat/military	USA
Rohr et al. (2021) [67]	Suicidal ideation	Psychiatric inpatients with PTSD	2822	CS	Mixed	USA
Livingstone et al. (2021) [68]	Substance use	Veterans	1243	L	Combat/military	USA

*CS*, cross-sectional; *L*, longitudinal; *NA*, not applicable

## Genetic and Epigenetic Factors

Eight studies were identified with relevant genomic evidence. In the largest sex-stratified genetic study of PTSD to date, a genome wide association study (GWAS) of a multi-ethnic cohort (~ 30,000 PTSD cases and 170,000 controls) found significant single nucleotide polymorphism (SNP) heritability of PTSD in females, (*h*^*2*^_SNP_ = 0.10, *P* = 8.03 × 10^−11^), while male heritability was not different from zero (*h*^*2*^_SNP_ = 0.01, *P* = 0.63) [9]. However, sex differences in heritability differed among subsets of data, which were attributed to diverse populations with differing traumatic experiences. For example, a higher prevalence of combat trauma among males *versus* females may explain the observed lower heritability estimates in males overall. Additionally, sex differences in specific risk loci were identified, with an additional three independent genome-wide significant loci found in men, but not women.

Genome wide gene-by-environment interaction studies (GEWIS) take a hypothesis-free approach to assessing genetic interactions with environmental variables. Leveraging data from the UK Biobank cohort, one study conducted a multivariate GEWIS of suicidality [10]. Sex-stratified GWEIS identified one independent suicidality risk loci in females and three in males. All risk loci showed robust evidence for interaction with a PTSD diagnosis or a PTSD risk score across sexes. Additional driving environments differed between females and males, with female risk loci interacting with post-traumatic stress symptoms and male risk loci interacting with physically violent trauma.

Large scale transcriptomic approaches can be utilised to explore mechanisms through which genetic variants may exert effects. A study of post-mortem tissue from 143 individuals with PTSD compared to age and sex matched controls applied differential gene expression and network analysis of four prefrontal cortex subregions [11]. Sex-specific transcriptomic signatures were identified across all regions of the prefrontal cortex, and females with PTSD showed more differentially expressed genes relative to controls (69 modules) than their male counterparts (59 modules). In addition, GABAergic signalling genes including *ELFN1*, *SST*, and *PNOC* were significantly downregulated in females, but not males. In a second study of this sample, the authors conducted exploratory co-expression network analysis to assess PTSD-specific neuropeptide signalling pathways. A total of three PTSD-associated networks contained neuropeptide related genes: two in females only and one within the full sample [12].

We found two small studies examining sex-specific epigenetic modifications in PTSD. First, a candidate gene methylation study examined oxytocin receptor gene *(OXTR)* methylation levels in police officers with (*n* = 31) and without PTSD (*n* = 36) [13]. Evidence was found for elevated *OXTR* DNA-methylation in females with PTSD, suggesting decreased central and peripheral oxytocin functioning, whereas PTSD-status was not associated with DNA-methylation in males. Additionally, increased methylation observed in PTSD females was predictive of higher symptoms of anhedonia and amygdala reactivity in response to emotional faces, but these associations did not survive correction for multiple testing. Second, a study combining two civilian cohorts (*n* = 483, including 277 with lifetime PTSD) harnessed methylomic profiles from peripheral blood to differences in leukocyte composition (i.e. proportions of peripheral immune cell subtypes) in association with PTSD [14•]. Higher monocyte proportions were observed in males in association with lifetime PTSD, independent of current PTSD status, suggesting a trait like alteration. This effect was not observed in females, and PTSD was unrelated to other leukocyte proportions. Further research on methylomic profiles in women and men with PTSD will shed light on sex differences in peripheral immune-neuroimmune mechanisms.

The role of genetics in wider PTSD linked outcomes has also been explored. Transcriptome-wide association studies (TWAS) detect genes in which *cis-*regulated expression is associated with a trait of interest and explore the mechanisms by which risk variants exert effects. One recent PTSD TWAS used brain and non-brain transcriptomic imputation to impute genetically regulated gene expression using data from 70 ancestry-specific cohorts (29,539 PTSD cases, 166,145 controls) [15]. Two study-wide PTSD associations were identified, both of which were stronger in males than females. This included downregulation of *SNRNP35* in the dorsolateral prefrontal cortex in European cohorts and upregulation *of ZNF140* in whole blood in European military cohorts.

Finally, Muniz Carvalho et al. [16] combined GWAS summary statistics from large cohorts of European and African ancestry to estimate sex-specific genetic correlation of PTSD with multiple indices of body size and composition [16]. Causal inference methods were then applied to significant genetic overlaps to test for putative causal effects. Evidence was found for sex-specific causal networks that linked anthropometric traits to PTSD, and in general these effects were found to be stronger in females than males. In the European female sample, fourteen anthropometric traits (including those related to BMI and adiposity measures) were found to have a putative causal effect on PTSD. In contrast, no evidence of causality for anthropometric traits on PTSD was found in the European male sample.

To conclude, there are multiple lines of emerging evidence for sexual dimorphism in the genetic architecture of risk and resilience to PTSD. To date, studies have been relatively underpowered, and it is likely additional sex specific risk loci will be identified as sample sizes for GWAS increase. Currently there is limited evidence regarding epigenetic modifications between sexes in PTSD, and the initiation of large-scale epigenome-wide association studies is warranted.

## Other Biological Factors

### Brain Structure and Function

PTSD is often associated with anatomical and functional alterations in three main areas involved in threat neurocircuitry — the amygdala, hippocampus, and the anterior cingulate cortex (ACC), including longitudinal prediction of PTSD by lower hippocampal [17] and ACC volumes [18] measured shortly following trauma. We identified eleven recent neuroimaging studies that have examined sex differences in brain structure and function due to PTSD.

A single recent longitudinal study examined PTSD risk based on brain structure in females *versus* males (total *n* = 94) [19•]. Intriguingly, results indicated that *greater* right rostral ACC thickness 1-month post-trauma predicted PTSD symptom severity at 6 months in females, an effect not found in males. This effect was attenuated but still significant when adjusting for PTSD symptoms at the time of scan, trauma type, and exposure to prior traumatic events. Follow-up analyses showed that right rostral ACC was positively correlated with *avoidance* symptoms in females (*r*(27) = 0.37, *p* = 0.04) but not males (*r*(44) = 0.04, *p* = 0.79).

Two cross-sectional studies examined sex differences in cortical thickness in relation to PTSD. First, in a large multicentre study from the ENIGMA-PGC PTSD Consortium (total *n* = 3397), no PTSD diagnosis *x* sex interactions were detected in relation to cortical thickness [20]. In a follow-up study by the same group, it was suggested that it may be important to examine cortical regions within the context of structural networks and that cortical regions *are* affected, albeit not significantly, in a coordinated manner by PTSD. Females with PTSD had a greater mean structural covariance in cortical thickness based *atrophic* networks, indicating that regions most affected by PTSD are modified in a systematic way in females only [21].

Structural MRI in combination with machine learning was used to examine the possible effect of PTSD on brain predicted age difference (brain PAD; brain age − chronological age) [22]. Analysis of data from the ENIGMA-PGC PTSD Consortium (*n* = 2229; aged 18–69 years) revealed that males, but not females, with PTSD exhibited higher brain PAD, indicative of accelerated brain aging. However, this effect was further qualified by age, with PTSD being associated with higher brain-PAD relative to controls in younger males. Among older males, control versus PTSD status was unexpectedly associated with relatively higher brain aging. Given evidence that PTSD is associated with reduced lifespan [23], such observations warrant further examination.

Sex-based alterations in white matter (WM) organization have also been examined in relation to PTSD, but studies have been limited and have been inconclusive. However, two recent studies are consistent in reporting no sex differences in white matter tracts due to PTSD. In a recent large multi-cohort analysis of >3000 individuals, no sex effects were found on diffusion metrics of fibre tracts in PTSD patients, with disrupted WM organisation observed in the tapetum region of the corpus callosum among patients overall [24]. Similarly, another recent study which used a tract-profile quantification machine learning approach to evaluate WM microstructure also found no sex-based effect on diffusion metrics between non-comorbid patients with PTSD (*n* = 77) and trauma-exposed non-PTSD controls (*n* = 76) [25].

Since disruption of glutamatergic synaptic strength may be a neuroimaging marker of PTSD severity, one small study utilised [1 ^13^C]-acetate magnetic resonance spectroscopy (MRS) to examine glutamatergic synaptic strength (measured as the energy-per-cycle [EPC]) in the prefrontal cortex (*n* = 34) [26]. Patients with PTSD were found to have 28% reduction in prefrontal EPC relative to controls, indicating reduced prefrontal neurotransmission; however, no significant sex effects were detected.

Three studies used functional MRI to investigate sex-based differences in PTSD in brain network topological architecture [27] and resting-state functional connectivity (FC) of the hippocampal subfields [28] and amygdala subnuclei [29]. In the first study, comparing Chinese parents with PTSD following the loss of an only child (*n* = 51) to similarly bereaved parents with no PTSD (NPTSD, *n* = 93) and non-bereaved controls (*n* = 50), a PTSD *x* sex interaction was observed in the nodal centralities (efficiency and degree) of the right parahippocampus [27]. Using a graph-theory based approach, regional activation was lower in male PTSD parents, compared with NPTSD parents, but higher in female PTSD parents than in female NPTSD parents. The increase of nodal centralities in the parahippocampus in PTSD females may promote fear conditioning in non-threatening contexts while interfering with normal fear extinction processes, although additional studies are needed to test this hypothesis. In a follow-up study of the same cohort (total *n* = 135), lost only-child without PTSD female participants also displayed *lower* right hippocampal CA3 subfield and thalamus functional connectivity (RCA3-RT FC) relative to female patients with PTSD [28]. This implies that lower FC may be a protective factor among women who have lost their only child, as they do not go onto develop PTSD. The same effect was not found in men. In the third smaller study of US veterans (*n* = 38), sex differences were observed in basolateral amygdala (BLA) resting state connectivity, which were associated with self-report scores of depression, anxiety, and aggression [29]. However, conclusions with respect to PTSD are substantially limited by the fact that all participants had comorbid PTSD/MDD.

Finally, the effects of intranasal oxytocin — a potential treatment target for PTSD — were examined in one study to measure changes in brain connectivity in response to implicitly presented facial affect cues in individuals with and without PTSD (total *n* = 34) [30]. Although some evidence was found of sex differences in neural responses to oxytocin, these were unrelated to PTSD status.

In sum, neuroimaging studies of PTSD have been relatively small, particularly in examining neurobiological predictors for identification of individuals at future risk of PTSD. Due to the nature of trauma research, and without scans collected pre-trauma, it is also difficult to determine causal influences. Large multi-study findings have begun to reveal some null-findings regarding sex-effects, particularly in differences in brain structure, but the application of other sensitive techniques to assess tissue microstructure should be considered. More consistent effects have emerged with regards to sex differences in functional connectivity and which are often further associated with PTSD symptoms. Further study is needed to examine how neurotransmitter systems contribute to sex differences in stress reactivity.

### Immunological, Neuroendocrine, and Neuropeptide Responses

Early-after-trauma immunological and neuroendocrine changes have been examined as putative biomarkers of the development of PTSD [31]. Five studies were identified which examined sex-related immunological and neuroendocrine responses.

In a prospective study of trauma survivors recruited from an emergency department (*n* = 376), immediate inflammatory responses were a significant mediator in the relationship between sex and probability of showing a *“non-remitting*” PTSD trajectory over 12 months [32•]. Specifically, an indirect pathway from sex to PTSD was identified, whereby men who had higher inflammatory cytokine levels ~ 3 h after trauma (i.e. greater inflammation) had a lower risk of developing PTSD symptoms over time. In post hoc analyses, this indirect effect was contingent upon oestradiol concentrations, a sex steroid known to impact pro-inflammatory cytokines, with higher levels of oestradiol being protective against the probability of developing PTSD in men. In the same sample, oestradiol, testosterone, and cortisol concentration did not predict non-remitting PTSD risk in either sex, while progesterone predicted probability of developing non-remitting PTSD in men only. The number of participants with non-remitting PTSD in the sample was small, meaning replication of these observations is desirable.

The second study accounted for circadian profiles whilst examining PTSD in relation to inflammation, given that sleep disturbances and cytokine elevations are linked [33]. Overnight levels of pro-inflammatory cytokines, including interleukin—6 (IL-6) and tumour necrosis factor- α (TNF-α), were measured in patients with chronic PTSD (*n* = 44) and matched controls (*n* = 49) while recording sleep polysomnography (PSG). Surprisingly, cytokine levels were not higher in those with PTSD at any single time point. However, overnight TNF-α *trajectories* differed by sex, such that TNF-α peaked in men with PTSD, but not women with PTSD, at the end of the sleep cycle, suggesting sex differences in the time course of pro-inflammatory overnight activity in PTSD. Given that the sample consisted of physically healthy, younger adults with less severe PTSD symptoms, replication in a more generalizable sample is warranted.

Fear conditioning and fear extinction are strongly regulated by circulating reproductive hormones (e.g. oestrogen). The third study examined the effect of sex, PTSD severity, and commonly used hormonal contraceptives (HC) on these processes, in trauma-exposed men and women with and without full or partial PTSD (total *n* = 81). Women on synthetic oestradiol in the form of HC demonstrated enhanced acquisition of fear conditioning and enhanced extinction of fear as compared with women not on HC and men [34]. No PTSD effects were identified. Nevertheless, as fear extinction is a foundational mechanism of exposure-based treatment for PTSD, these findings have potential implications for the treatment of PTSD in women with low circulating oestradiol levels.

Finally, the functioning of the HPA axis and hypothalamic neuropeptides (e.g. neuropeptide Y and oxytocin) has been examined in two recent studies. First, lower oxytocin levels were found in PTSD patients (*n* = 26) relative to controls (*n* = 26), with no sex differences reported in this small study [35]. Second, while a strong association was found between female sex and higher NPY levels, sex specific NPY differences in individuals with PTSD have not yet been established [36].

### Role of Sleep

Sleep disruption in PTSD has been documented in previous studies to differ by sex, with greater rapid eye movement sleep (REM) displayed in women with PTSD compared to controls [37, 38]. However, the mechanisms underlying these sex differences are poorly understood.

A single recent study was identified which examined the role of sex in relation to the sleep-emotion processing relationship [39]. Using a fear-potentiated startle and nap sleep protocol in trauma-exposed participants with varying PTSD severity (total *n* = 46), higher safety learning predicted increased REMS and increased REMS predicted more rapid extinction learning overall. However, the safety signal learning effect on subsequent REM was explained by a robust effect in males only. Given that REM sleep predicted greater extinction learning and is critical for sleep-dependent emotional information processing suggests that males may benefit from the increased REM response, but females do not. However, the potential for type 1 and type II errors should be considered given the sample size for considering interaction effects.

## Rates of Recovery and Response to Treatment

Sixteen recent studies have examined sex differences in recovery, both observationally and in the course of treatment for PTSD.

Four studies examined sex differences in the natural recovery of symptoms. In their meta-analysis of longitudinal observational studies including 16,484 adults, Diamond and Airdrie [40•] identified female sex as being associated with higher initial levels of PTSD following trauma, but also with stronger recovery in the first 6 months. Sex explained 36.8% of the variance in PTSD prevalence change between 1 and 6 months, with this effect being attenuated but still observable when adjusting for initial symptom levels. Consistent with these observations, a latent class analysis of emergency department patients (*n* = 554) identified resilient, recovery, chronic symptoms and delayed onset PTSD trajectories over a 12-month follow-up period, with the recovering trajectory being more prevalent among women and delayed onset of PTSD being more prevalent in men [41].

Two of these studies have examined PTSD recovery in more chronic populations. First, a cross-sectional analysis of adults with lifetime PTSD from a nationally representative US survey (*n* = 1997) found extremely similar rates of remission based on the absence of past-year diagnosis for women (25.3%) and men (24.3%) [42]. Duration of PTSD could not be established in this dataset. Second, a study of US veterans used an accelerated longitudinal design using 5-year follow-up coupled with reported time since index trauma to estimate recovery trajectories across a 20-year window [43]. There was no evidence of sex differences in either overall PTSD prevalence or recovery trajectories. The study oversampled for veterans with a PTSD diagnosis (75% of sample), which likely captured a relatively chronic sample and may have diminished capacity to capture sex differences.

Sex differences in response to interventions were also reported in four studies. In the first study of 10,234 US veterans, compared to men, women had a greater likelihood of clinically meaningful improvement with fewer sessions of prolonged exposure (PE) although the effect was small (*HR*: 1.13; 95% CI: 1.02–1.25) [44]. Median number of PE sessions received in this study was 2, with 40.5% showing clinically meaningful improvement. The second study of US veterans focused on those receiving at least 8 sessions of cognitive processing therapy (CPT) or PE (*n* = 9711). Here, adjusted analyses found a 4.4% greater decrease in women’s symptom scores compared to men for CPT only (mean symptom reduction across treatments approximately 17%) [45•]. A third US veteran study examined intensive treatments for PTSD, including inpatient, day hospital, and residential programs, with a mean length of stay of 42 days. Analysis of data for 3370 veterans found greater symptom reduction in females versus males although the effect size was small (Cohen’s *d* = − 0.29) [46]. Finally, in a study of 948 patients diagnosed with PTSD following mixed traumas, female sex was associated with greater response to care received in Danish public outpatient clinics [47]. Care packages could vary in terms of the type of psychotherapy provided, the level of training of the provider, and the delivery mode, and the outcomes in the study must be considered in the context of low rates of PTSD remission overall (18.8%).

Additional evidence suggests that considering wider therapeutic outcomes may be important during the course of treatment. Glassman and colleagues examined change in quality of life (QOL) following cognitive processing therapy in two randomized controlled trials with all female (*n* = 126) and all male (*n* = 125) participants [48]. In the female sample, reductions in depressive symptoms were strongly associated with QOL improvements [*B* = − 1.15 (95% CI: − 1.71, − 0.60), whereas reductions in trait anger predicted increased QOL in males [*B* = − 0.55 (95% CI: − 0.90, − 0.19)] (reductions in PTSD were not associated with QOL change in either sex). Although these findings highlight potentially important clinical targets for PTSD interventions that differ by sex, conclusions are limited by the fact that the male sample comprised of veterans, whereas veterans formed a minority of female participants.

Three studies have examined the effect of biological intervention in recovery by sex. One record-based review found no evidence of sex effects in symptom reduction among 6879 US veterans following an adequate course of medication [49]. However, mean reduction in symptom scores in this population was modest (approximately 10%). By contrast, a multi-country study of 390 adults with PTSD to mixed traumas found both robust symptom reductions over a 12-week course of paroxetine or sertraline (mean symptom score decline of 48%, 58.5% responders) and evidence of sex differences in recovery, with females showing stronger recovery than males [50]. Finally, an analysis of a mixed trauma population (total *n* = 327) treated with stellate ganglion blockage (i.e. using local anaesthetic proximal to the stellate ganglion to achieve inhibition of sympathetic activity to the brain) found no evidence of sex differences in response, with 69.7% of patients achieving at least a 20 point symptom reduction on the PTSD Checklist overall [51].

A final area of investigation in terms of treatment approaches has been to consider sex differences in engagement and provision, with four studies identified. In 7218 US veterans with PTSD, females were more likely than males to initiate individual psychotherapy within 6-months of diagnosis (52.3% versus 40.1% of men; *OR* 1.37) and more likely to complete an adequate course (17.7% versus 10.8%; *OR* 1.41) [52]. Notably, the sex difference in initiation of treatment was evident at older but not younger ages. In addition, a longitudinal study of 1200 US veterans (704 women; three assessments conducted across 8 months) found that females had higher PTSD symptoms and also higher levels of mental health service use than males (34.5% for female versus 27.6% for male veterans). Moreover, both sexes reported poorer intimate relationship functioning in association with higher PTSD symptoms, but poorer relationship functioning at T2 provided an indirect pathway from T1 symptoms to T3 treatment access in males only [53].

Conflicting findings have also been reported. A study of 95 veterans and first responders found that females were more likely to drop out of a residential treatment for PTSD (*r* = 0.270; overall dropout rate 59%). The number of females in this sample was small (*n* = 13) [54]. Finally, an analysis of prescribing patterns for 877,785 US veterans receiving treatment for PTSD in 2019 (13.5% women) found that women were slightly more likely than men to receive antidepressants recommended for PTSD (adjusted *OR* = 1.07), but were also more likely to receive medications recommended *against* use for PTSD, including benzodiazepines (adjusted *OR* = 1.62), anticonvulsants (a*OR* = 1.41), and antidepressants recommended against use for PTSD (adjusted *OR* = 1.26), in analyses adjusted for comorbidities and other confounders [55•].

In sum, recent studies of recovery from PTSD provide relatively robust evidence of stronger female versus male recovery in the first 1–2 years post-trauma, but not over longer timescales. We found consistent evidence of greater PTSD symptom reduction in women versus men with PTSD following psychotherapy for PTSD, even accounting for factors such as baseline severity. Where reported, effect sizes for sex were small, and the majority of treatment outcome studies examining sex differences have been conducted in veteran samples, with a dearth of data in civilian samples. By contrast, limited evidence was found for sex differences in responses to biological interventions. There was some evidence of greater female engagement with treatment but findings here were mixed.

## Medical and Psychiatric Comorbidities

PTSD is associated with non-communicable diseases, including cardiovascular disease (CVD), type-2 diabetes [56, 57], and dementia [58••]. Recent evidence from 10 studies provide insight into possible sex differences underpinning these associations.

In a 13-year prospective study of nearly 1 million young-and middle-aged US veterans, PTSD was a stronger risk factor for ischemic stroke in men relative to women [59] even when adjusting for established stroke risk factors, psychiatric comorbidities, and healthcare utilisation (adjusted *HR*: 0.63; 95% CI, 0.47–0.86). Three studies have directly assessed the mechanisms by which PTSD may interact with sex in CVD risk. In a prospective study, sex differences in multiple domains of autonomic functioning were reported in a recently traumatised sample (total *n* = 192) [60]. Two weeks post-trauma, men were more likely to meet the criteria for hypertension (45% versus 21%) compared to women, reflected by higher systolic, but not diastolic, BP. In addition, during the extinction phase of a fear conditioning paradigm, HR was significantly higher in women with PTSD (*M* = 78.5) compared to men with PTSD (*M* = 67.7), whereas high frequency heart rate variability was significantly lower in women with PTSD (*M* = 5.41) compared to men with PTSD (*M* = 6.49), indicating lower parasympathetic control in women during fear learning. In contrast, a cross-sectional study of nearly 3000 trauma-exposed men and women [61] reported that men with PTSD had a *lower* risk of hypertension when measuring each component of metabolic syndrome, which survived adjustment for additional factors such as cancer, angina, stroke, and thyroid disease (adjusted *OR* = 0.54, 95% CI = 0.31–0.92). For women, PTSD was associated with metabolic syndrome overall (adjusted *OR* = 1.53, 95% CI = 1.01–1.95) and the prevalence of the high-density lipoprotein cholesterol component (adjusted *OR* = 1.98, 95% CI = 1.04–2.12), indicating that the relationship between PTSD and metabolic syndrome may be confined to women. Finally, a small cross-sectional study (*n* = 61) found sex differences in the association between *subclinical* PTSD symptoms and subclinical atherosclerosis in young adults [62]. In men, PTSD symptom severity was associated with increased aortic stiffness, indicative of vascular damage which may contribute to the increased risk of CVD. In contrast, women with greater symptoms were more likely to have peripheral vasomotor dysfunction, which can contribute to conditions such as hypertension, diabetes, and hypercholesterolemia [63].

In the first meta-analysis of global longitudinal evidence on PTSD and dementia risk (*k* = 8, *n* = 1,693,678), the authors found that people with PTSD had a 61% increased risk of developing all-cause dementia [58••]. This risk was higher in studies that included ≥ 50% females (pooled hazard ratio *[HR]* = 1.97, 95% CI 1.25–3.11) compared with studies with < 50% female participants (pooled *HR* = 1.62, 95% CI 1.43–1.85). In addition, a population-based cohort study based in Israel examined sex differences in Parkinson’s disease (PD) risk in patients with (*n* = 8336) and without (*n* = 8336) PTSD [64]. Overall, patients with PTSD had a 48% increased risk for developing PD (adjusted *HR* 1.48 [95% CI, 1.10–1.99]). Sex and age stratified analysis indicated that the risk of developing PD is greater in male patients who received a diagnosis of PTSD at the age of 72 years or older (adjusted *HR* 1.95 [95% CI, 1.16–3.28]).

Given observations that PTSD is associated with immune dysregulation and inflammation, it is unsurprising that a bidirectional relationship with infectious disease has also been reported. In a longitudinal nationwide cohort study of all residents of Denmark diagnosed with PTSD between 1995 and 2011 (*n* = ~ 30,000), the association between PTSD and some infections (e.g. urinary tract and viral infections) was found to be stronger among women, whereas other associations were stronger among men (e.g*.* skin infections) [65].

Three studies have also provided sex-specific differences in psychological comorbidities in association with PTSD. In a subsample of 9447 US service members with PTSD, 83% of individuals with PTSD had at least one comorbid condition [66]. Women with PTSD were more likely to have depressive, generalised, or other anxiety disorders, with largest effect sizes for eating (*OR*: 12.6, 95% CI 7.9–20.1) and personality disorders (*OR*: 3.0, 95% CI 2.6–3.4). In contrast, men with PTSD were more likely to have a diagnosis of PTSD with comorbid alcohol and drug use disorders, with traumatic brain injury almost six times more prevalent and insomnia over twice as common in men compared to women. Given that sleep disturbances have a more prominent effect on suicidal ideation in men with PTSD compared to women (total *n* = 822 inpatients) suggests that it may be especially important for men to undergo empirically supported treatments for insomnia [67]. Finally, a longitudinal study of US veterans (*n* = 1243) found evidence that PTSD symptoms predicted future illicit drug use problems among men, whereas drug use problems predicted future PTSD symptom severity among women [68]. Thus, it was concluded PTSD severity should be monitored as a risk factor for future drug use in men, whereas women’s drug use should be monitored as a particular risk factor for the increasing severity of PTSD symptoms.

Overall, these studies present a complex picture of possible sex-specific effects in the PTSD-disease pathway. Men appear to have heightened risk for cardiovascular disease possibly through PTSD-related changes to blood pressure, whereas women may be more at risk for dementia and metabolic syndrome. Further exploration that includes potential differential biological and behavioural (e.g. health risk behaviours) pathways is warranted.

## Conclusions

Various sex-related biological factors contribute to the differences in PTSD risk, clinical presentation, comorbidity, treatment response, and retention in care. Over the last 3 years, evidence has emerged on specific biological mechanisms that may be driving sex-related patterns in individuals with PTSD. The majority of studies identified in this review, however, were conducted in high-income countries; biological contributors and consequences of adult PTSD will additionally need to be examined in low-to-middle income country settings [69]. Few studies have also been primarily designed, and statistically powered, to investigate sex differences in PTSD. Future studies should adopt an integrated, multi-level biological system approach to investigate the association of epigenetic, genetic, and gene expression signatures in PTSD (e.g. genes underpinning hypocortisolaemia in PTSD), biochemical assays of circulating sex hormones and other HPA axis markers, coupled with assessments of clinical presentation, course, and treatment response and recovery in females and males. This will be the next step in parsing out functional mechanisms that could represent sex-selective targets for therapeutic interventions in PTSD.
